# Comparison of single-shot EPI and multi-shot EPI in prostate DWI at 3.0 T

**DOI:** 10.1038/s41598-022-20518-8

**Published:** 2022-09-27

**Authors:** Tsutomu Tamada, Ayumu Kido, Yu Ueda, Mitsuru Takeuchi, Akihiko Kanki, Jaladhar Neelavalli, Akira Yamamoto

**Affiliations:** 1 Department of Radiology, Kawasaki Medical School, 577 Matsushima, Kurashiki, Okayama 701-0192 Japan; 2 Philips Japan, Tokyo, Japan; 3 Department of Radiology, Radiolonet Tokai, Nagoya, Japan; 4 Philips India, Bangalore, India

**Keywords:** Cancer, Medical research, Urology

## Abstract

In prostate MRI, single-shot EPI (ssEPI) DWI still suffers from distortion and blurring. Multi-shot EPI (msEPI) overcomes the drawbacks of ssEPI DWI. The aim of this article was to compare the image quality and diagnostic performance for clinically significant prostate cancer (csPC) between ssEPI DWI and msEPI DWI. This retrospective study included 134 patients with suspected PC who underwent 3.0 T MRI and subsequent MRI-guided biopsy. Three radiologists independently assessed anatomical distortion, prostate edge clarity, and lesion conspicuity score for pathologically confirmed csPC. Lesion apparent diffusion coefficient (ADC) and benign ADC were also calculated. In 17 PC patients who underwent prostatectomy, three radiologists independently assessed eight prostate regions by DWI score in PI-RADS v 2.1. Anatomical distortion and prostate edge clarity were significantly higher in msEPI DWI than in ssEPI DWI in the three readers. Lesion conspicuity score was significantly higher in msEPI DWI than in ssEPI DWI in reader 1 and reader 3. Regarding discrimination ability between PC with GS ≤ 3 + 4 and PC with GS ≥ 4 + 3 using lesion ADC, AUC was comparable between ssEPI DWI and msEPI DWI. For diagnostic performance of csPC using DWI score, AUC was comparable between msEPI DWI and ssEPI DWI in all readers. Compared with ssEPI DWI, msEPI DWI had improved image quality and similar or higher diagnostic performance.

## Introduction

Prostate multiparametric MRI commonly consists of T2-weighted imaging (T2WI), diffusion-weighted imaging (DWI), and dynamic contrast-enhanced MRI (DCE-MRI). In patients with elevated prostate-specific antigen (PSA) levels, the combination of multiparametric MRI and MR-guided prostate biopsy has higher diagnostic performance for detection of clinically significant prostate cancer (csPC) eligible for curative therapies such as radical prostatectomy are indicated compared with standard systematic prostate biopsy^1–4^. Multiparametric MRI, especially DWI, has already made a strong contribution to the accumulation of research results with regard to the detection and localization of primary csPC and local recurrence, assessment of tumor aggressiveness by such as Gleason score (GS) and Gleason grade, local staging, active surveillance, and standardization of prostate MRI diagnosis (Prostate Imaging Reporting and Data System [PI-RADS])^5–17^. Furthermore, in recent years, numerous studies regarding biparametric MRI, which does not include DCE-MRI, have reported comparable diagnostic accuracy between biparametric MRI and multiparametric MRI for detecting csPC^18–23^. Therefore, DWI requires high lesion detectability and stable image quality with minimal artifacts.

DWI with single-shot echo-planar imaging (ssEPI) is the most commonly used in routine clinical practice due to its high signal-to-noise ratio (SNR), rapid acquisition, and insensitivity to motion^7,16,17^. Its main drawbacks are distortion due to B0 inhomogeneity and blurring due to T2* attenuation^11,24–27^. Although parallel imaging has enabled dramatic reductions in distortion and blurring, these continue to affect ssEPI DWI, especially in the presence of rectal gas and in high-resolution images, respectively^28,29^. Multi-shot EPI (msEPI) DWI, in which k-space data are acquired in multiple excitations, can reduce distortion and blurring because the effective echo spacing and echo train length are shorter. However, msEPI DWI is sensitive to motion during diffusion encoding between shots, causing phase error that results in severe artifacts^30,31^. MsEPI DWI, which is termed image reconstruction using the image-space sampling function (IRIS) in Philips systems, can minimize motion effects between shots by implementing two-dimensional (2D) navigator echo, and provide robust image quality even in the presence of tissue motion. Therefore, msEPI DWI enables a reduction in image distortion and blurring compared with conventional ssEPI DWI. Other msEPI DWI techniques related to IRIS are readout segmentation of long variable echo trains (RESOLVE) and multiplexed sensitivity encoding (MUSE)^32,33^. In contrast to IRIS, which is an interleaved, phase-segmented technique, RESOLVE is a read-out segmented, navigator-based multishot DWI technique. MUSE is similar to IRIS in that it is also a phase-segmented, interleaved multi-shot technique. However, it does not use an additional navigator. Each shot is reconstructed individually using parallel imaging and the respective phase data are used for shot-to-shot phase correction. To our knowledge, this is the first report of IRIS applied to the prostate. The purpose of this study was to compare the image quality and diagnostic performance for detecting csPC between ssEPI DWI and msEPI DWI under the same acquisition time, to investigate the clinical feasibility of msEPI DWI for prostate DWI.

## Materials and methods

This study was approved by the ethics committee of Kawasaki Medical School (Application Number: 5450-00) and was conducted in accordance with principles of the 1964 Helsinki Declaration and its later amendments or comparable ethical standards. The requirement to obtain informed consent for this retrospective study was waived.

### Study population

We initially identified the records of 175 consecutive male patients with elevated PSA levels or suspected PC who underwent 3.0 T prostate multiparametric MRI between October 2020 and June 2021. Of these patients, 41 were excluded for the following reasons: severe motion artifact on DWI (*n* = 3); incomplete DWI examination with no msEPI DWI acquisition (*n* = 27); previous prostatectomy (*n* = 3); and duplicate scans (n = 8). Thus, 134 patients (age range, 49–92 years; mean age, 71.4 years) were included in the study. Median PSA level at the time of initial MRI was 6.58 ng/mL (PSA range, 0.61–172.1 ng/mL). Of the 134 patients, 92 underwent subsequent MR-guided biopsy for lesions suggestive of PC on multiparametric MRI including T2WI, ssEPI DWI, and DCE-MRI, of which 55 were histopathologically confirmed as csPC. The mean interval between MRI examination and MR-guided biopsy was 46 ± 34 days (range, 1–176 days; median, 35 days). Seventeen of the patients underwent radical prostatectomy after MR-guided biopsy. Eleven patients had undergone treatment by radiotherapy (*n* = 7), hormonal therapy (*n* = 1), or both radiotherapy and hormonal therapy (*n* = 3) for PC at the time of the MRI examination. All lesions were diagnosed as PC by MR-guided biopsy or prostatectomy-derived histopathology.

### MR-guided biopsy

The UroStation system (Koelis; Grenoble, France) was used for the MR-guided biopsy procedure. The system has elastic image fusion technology, real-time three-dimensional (3D) organ-tracking technology, and a computer workstation (Koelis) for segmentation of the prostate and the lesion. The target lesions for MR-guided biopsy were determined by fellowship-trained abdominal radiologists at the time of multiparametric MRI examination using PI-RADS v2, PI-RADS v2.1, and conventional overall multiparametric MRI assessment^16,17,34^. In the targeted biopsy, at least two cores were obtained from each MRI-targeted lesion. The operator determined the need for additional cores based on lesion size, lesion location, and confidence in targeting accuracy.

### Histopathologic analysis

All specimens obtained from MR-guided biopsy and radical prostatectomy were stained with hematoxylin–eosin. Radical prostatectomy specimens underwent standard step sections at 4- to 6-mm intervals. All specimens were reviewed and diagnosed by board-certified attending pathologists. Tumor GSs were assigned in accordance with the 2014 International Society of Urological Pathology Modified Gleason Grading System^35^. Regarding tumor GS in 55 PCs, prostatectomy GS was indicated in patients who underwent radical prostatectomy (*n* = 17), and biopsy GS in those who underwent MR-guided biopsy only (*n* = 38). Tumor size was calculated from multiparametric MR images, most commonly T2WI. CsPC was defined as GS 6 and tumor size 0.5 mL or greater (tumor diameter 8 mm or greater) or as GS 7 or greater and tumor diameter 5 mm or greater. If a patient had multiple PCs, the index lesion was defined as that with the highest GS and largest size among the csPCs.

### Imaging technique

All prebiopsy prostate MRI examinations were performed on 3.0 T MRI scanners (Ingenia 3.0 T CX Quasar Dual or Ingenia Elition 3.0 T; Philips Healthcare, Best, the Netherlands) equipped with a 32-channel digital coil for signal reception. Immediately before the examination, scopolamine butylbromide or glucagon was administered intramuscularly to reduce intestinal peristalsis. The MR imaging protocol consisted of the following sequences: axial T2WI, axial DCE-MRI, axial DWI, coronal T1-weighted imaging (T1WI), and 3D T2WI. We did not assess coronal T1WI or 3D T2WI in this study. Axial turbo spin echo T2WI was acquired with the following parameters: repetition time (TR), 7257 ms; echo time (TE), 95 ms; slice thickness, 3 mm; no intersection gap; field of view (FOV), 200 × 200 mm^2^; matrix, 352 × 277; in-plane resolution, 0.57 × 0.72 mm^2^; parallel imaging factor, 1.4. DCE-MRI began simultaneously with the start of intravenous injection of a gadolinium-based contrast medium at 0.1 mmol/kg body weight at a rate of 1.5 mL/s (gadobutrol; Gadovist; Bayer Schering Pharma, Osaka), followed by a 30-mL saline flush at the same rate as the contrast medium injection. For DCE-MRI, 3D T1-weighted gradient echo sequence with fat-suppression (TR, 4.0 ms; TE, 1.44 ms; slice thickness, 2 mm; no interslice gap; FOV, 350 × 317 mm^2^; matrix 288 × 230; in-plane resolution, 1.22 × 1.38 mm^2^; parallel imaging factor, 2.1) was performed dynamically every 10 s for 180 s (18 phases) without breath-holding. The imaging parameters for ssEPI DWI and msEPI DWI are listed in detail in Table 1. The same acquisition time was set for both ssEPI DWI and msEPI DWI (4 min 24 s). In msEPI DWI, multiple excitation is performed in the phase direction. Apparent diffusion coefficient (ADC) maps were calculated using b-values of 0 and 1500 s/mm^2^ by non-Gaussian, mono-exponential fitting model.

**Table 1:** DWI imaging parameters.

	ssEPI DWI	msEPI DWI
Field of view (mm)	300 × 300	300 × 300
Number of slices	30	30
TR/effective TE (ms)	5500/62	5500/62
Acquired voxel size (mm)	3.13 × 3.19 × 3.00	3.13 × 3.26 × 3.00
Reconstruction voxel size (mm)	1.17 × 1.17 × 3.00	1.17 × 1.17 × 3.00
Number of shots	1	2
EPI factor	47	23
SENSE factor	2	2
Partial Fourier factor	0.817	NA
b-values (s/mm^2^)	0, 1500	0, 1500
Number of signal averages	2 (b0), 15 (b1500)	2 (b0), 7 (b1500)
Acquisition time	4 min 24 s	4 min 24 s

### Image analysis

All image analysis was performed using a dedicated workstation (Synapse EX; Fujifilm Corporation, Tokyo, Japan). Three fellowship-trained radiologists with 9 years (Reader 1), 13 years (Reader 2), and 23 years (Reader 3) of experience in prostate MRI independently assessed the DW images of each patient.

First, in qualitative analysis, we used three types of indices: anatomical distortion of prostate, prostate edge clarity, and lesion conspicuity score for pathologically confirmed csPC. The radiologists assessed the qualitative indices in the DW images visually using a 4-point grading scale.

The 4-point grading scale of each index was defined as follows:

Anatomical distortion, 1 = severe; 2 = moderate, 3 = mild; 4 = none;

Prostate edge clarity, 1 = very poor; 2 = poor, 3 = moderate; 4 = good;

Lesion conspicuity score, 1 = invisible for surrounding normal site; 2 = slightly high, 3 = moderately high; 4 = very high.

Anatomical distortion and prostate edge clarity were assessed for all 134 patients with and without csPC, and lesion conspicuity score was assessed for an index lesion in 55 patients with pathologically confirmed csPC by MR-guided biopsy. The index lesions for each patient were shown to each radiologist using a PowerPoint file that displayed the DW images of the lesion targeted for MR-guided biopsy. The ssEPI DWI images and msEPI DWI images were assessed separately, with a time interval of > 4 weeks between the assessments.

Second, quantitative analysis of the DW images was performed by two radiologists (Readers 1 and 3) in consensus. We measured four types of quantitative indices: SNR, contrast-to-noise ratio (CNR), mean ADC (× 10^–3^ mm^2^/s) of the lesion (lesion ADC), and surrounding benign prostatic parenchyma (benign ADC).

SNR and CNR were defined as follows:

SNR = the signal intensity (SI) (mean) of benign prostatic parenchyma (benign prostate SI)/internal obturator muscle SI (standard deviation [SD]).

CNR = (lesion SI [mean] − benign prostate SI [mean])/internal obturator muscle SI [SD]).

**Figure 1:**
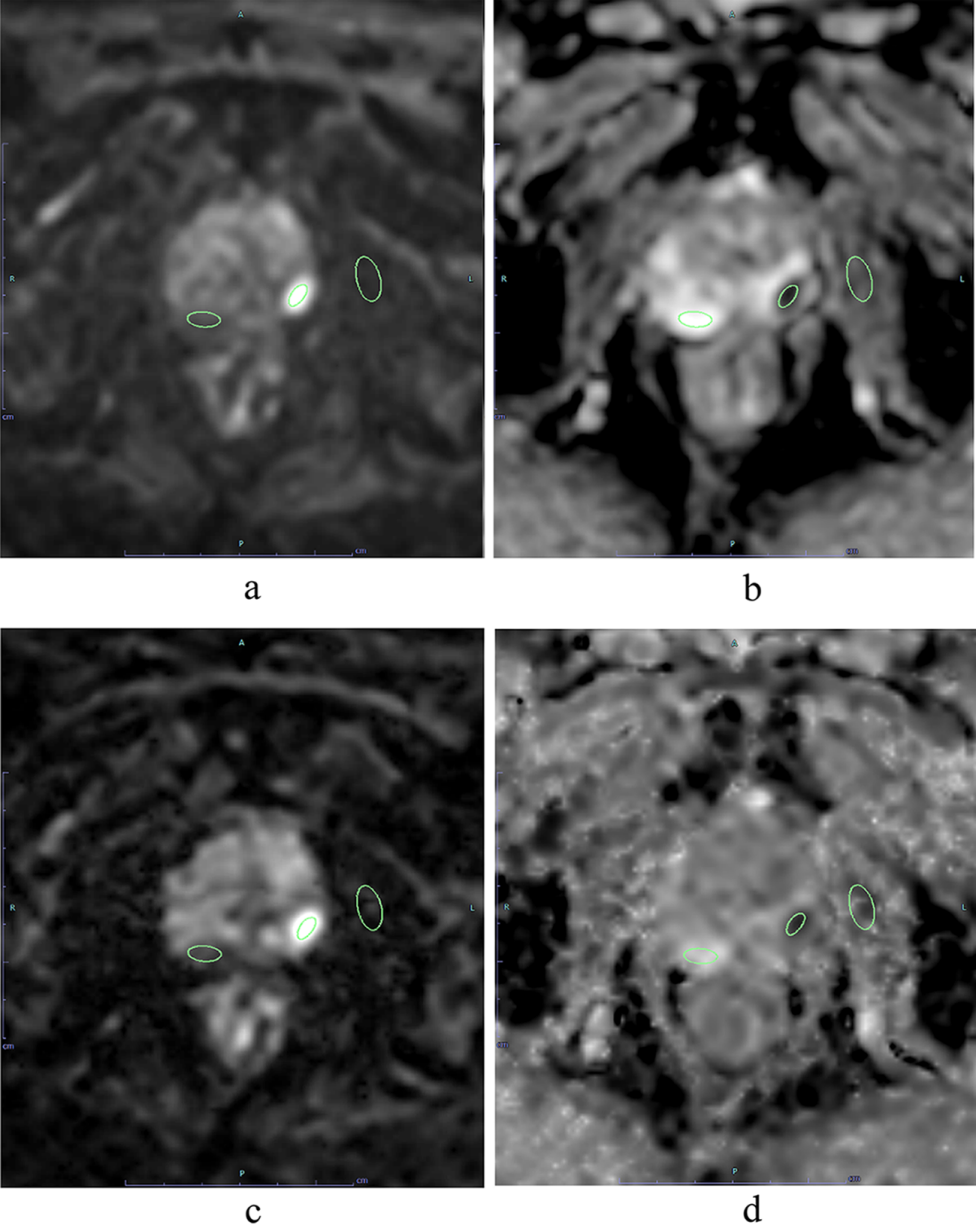
Region of interest (ROI) placement for signal intensity and ADC measurements of prostate cancer region in left peripheral zone and benign prostatic region in right peripheral zone and for signal intensity measurement of left internal obturator muscle region. The ROIs for PC region, benign prostatic region, and internal obturator muscle region in one of these four MR images was copy-and-pasted into the other three MR images [ssEPI DWI (**a**), ssEPI DWI ADC map (**b**), msEPI DWI (**c**), and msEPI DWI ADC map (**d**)].

SNR was calculated for all 134 patients with and without csPC and CNR, lesion ADC, and benign ADC were calculated for the 55 patients with pathologically confirmed csPC by MR-guided biopsy. The SI and ADC of the index lesion and benign prostatic region and the SI of the internal obturator muscle region were measured in each patient using a region-of-interest (ROI) placement technique (Fig. 1). The ROIs were as large a circle or oval as possible. The radiologists reviewed the four types of MR images including ssEPI DWI, ssEPI DWI ADC map, msEPI DWI, and msEPI ADC map in parallel on a PACS monitor to minimize positioning gaps in ROI placement. The ROIs for PC regions and benign prostatic regions in one of these four MR images was copy-and-pasted into the other three MR images of each subject (Fig. 1). In some patients, there were some mis-registration between ssEPI and msEPI, so visual fine adjustment was performed after copy-and-pasting the ROI. The ROIs for SI and ADC measurements of the PC lesion encompassed the entire area of the slice passing through the center of the lesion.

The ROIs for benign prostatic regions were placed at a location corresponding to benign prostatic parenchyma of the peripheral zone, based on histopathological and radiological findings including MR-guided biopsy, prostatectomy, and multiparametric MR images. The ROIs in benign prostatic regions were placed to avoid the periprostatic venous plexus, neurovascular bundle, and urethra. For the internal obturator muscle, the radiologists placed an ROI on the region on the left or right side that showed uniform signal intensity relative to T2WI.

Third, the diagnostic performance of ssEPI DWI and msEPI DWI for csPC detection in 17 PC patients who underwent prostatectomy was evaluated 4 weeks after the above qualitative and quantitative analysis. The csPCs included all lesions in prostatectomy specimens. The same three radiologists independently assessed eight regions (six peripheral zone: right apex, right middle, right base, left apex, left middle, and left base; and two transition zones: right transition zone and left transition zone) using DWI score in PI-RADS v 2.1 (136 regions in total) (Table 2)^17^. Each radiologist assigned scores of 1–5 for DWI for each region using DW images and ADC maps. The study coordinator prepared an Excel file showing the DW image numbers of each region referenced to axial T2WI to avoid recognition differences of prostate zonal anatomy among readers. Readers were aware of patient age, but were blinded to PSA level and the histopathology results. The ssEPI DWI and msEPI DWI were assessed separately, with a time interval of > 4 weeks between the assessments.

**Table 2:** Scoring system of diffusion-weighted imaging for assessment of transition zone and peripheral zone in PI-RADS v2.1.

Score	PI-RADS v2.1
1	No abnormality (i.e., normal) on ADC map and DWI
2	Linear/wedge-shaped hypointense on ADC and/or linear/wedge-shaped hyperintense on high b-value DWI
3	Focal (discrete and different from the background) hypointense on ADC and/or focal hyperintense on high b-value DWI; may be markedly hypointense on ADC or markedly hyperintense on high b-value DWI, but not both
4	Focal markedly hypointense on ADC and markedly hyperintense on high b-value DWI; < 1.5 cm in greatest dimension
5	Same as 4 but ≥ 1.5 cm in greatest dimension or definite extraprostatic extension/invasive behavior

### Statistical analysis

We performed all statistical analyses using JMP v11.0.0 software (SAS, Cary, NC) and SPSS for Windows v24.0 software (SPSS, Chicago, IL). Wilcoxon signed rank test was used to compare anatomical distortion, prostate edge clarity, lesion conspicuity score, SNR, CNR, and lesion ADC between ssEPI DWI and msEPI DWI. For each of ssEPI DWI and msEPI DWI, Wilcoxon signed rank test was also used to compare between lesion ADC and benign peripheral zone ADC. Significant differences in the ADC values of ssEPI DWI and msEPI DWI between PC with GS ≤ 3 + 4 and PC with GS ≥ 4 + 3 were determined by Mann–Whitney U test^35,36^. For csPC detection ability in 17 patients with prostatectomy (136 regions), sensitivity, specificity, positive predictive value, negative predictive value, and accuracy when positive for PI-RADS v2.1 DWI score ≥ 3 were measured for ssEPI DWI and msEPI DWI in all readers. Sensitivity, specificity, and accuracy between ssEPI DWI and msEPI DWI for each reader were compared by the McNemar test. Diagnostic performance of ssEPI DWI and msEPI DWI for csPC detection in all readers was evaluated using receiver-operating characteristic (ROC) analysis. For each radiologist, comparison of the area under the curve (AUC) obtained from the ROC analysis between ssEPI DWI and msEPI DWI was performed using the Delong test. A value of *p* < 0.05 was considered statistically significant.

## Results

### Patients and diagnoses

In this study, the lesions of 55/92 patients who underwent MR-guided biopsy were histopathologically confirmed as csPC. Of the 55 lesions, 24 were located in the peripheral zone, 21 in the transition zone, and 10 in both the peripheral zone and transition zone. The distribution of GS of the 55 csPCs was as follows: GS = 3 + 3, *n* = 9; GS = 3 + 4, *n* = 22; GS = 4 + 3, *n* = 14; GS = 4 + 4, *n* = 6, and GS = 4 + 5, *n* = 4. The mean diameter of csPC was 16.2 mm ± 10.4 mm (range, 5.0–56.6 mm). Forty csPC lesions were found in the 17 prostatectomy specimens, including GS = 3 + 3 (*n* = 2), GS = 3 + 4 (*n* = 17), GS = 4 + 3 (*n* = 20), and GS = 4 + 5 (*n* = 1). Twenty-one lesions were located in the peripheral zone, 13 in the transition zone, and 6 in the peripheral zone and transition zone. They comprised one lesion in 5 patients, 2 lesions in 6 patients, 3 lesions in 4 patients, 5 lesions in one patient, and 6 lesions in one patient. Mean csPC diameter was 13.0 mm ± 6.2 mm (range, 5.2–28.3 mm). In the analysis of diagnostic performance using patients who underwent prostatectomy, 68/136 regions in 17 prostatectomy patients were csPCs.

### Assessment of image quality

**Figure 2:**
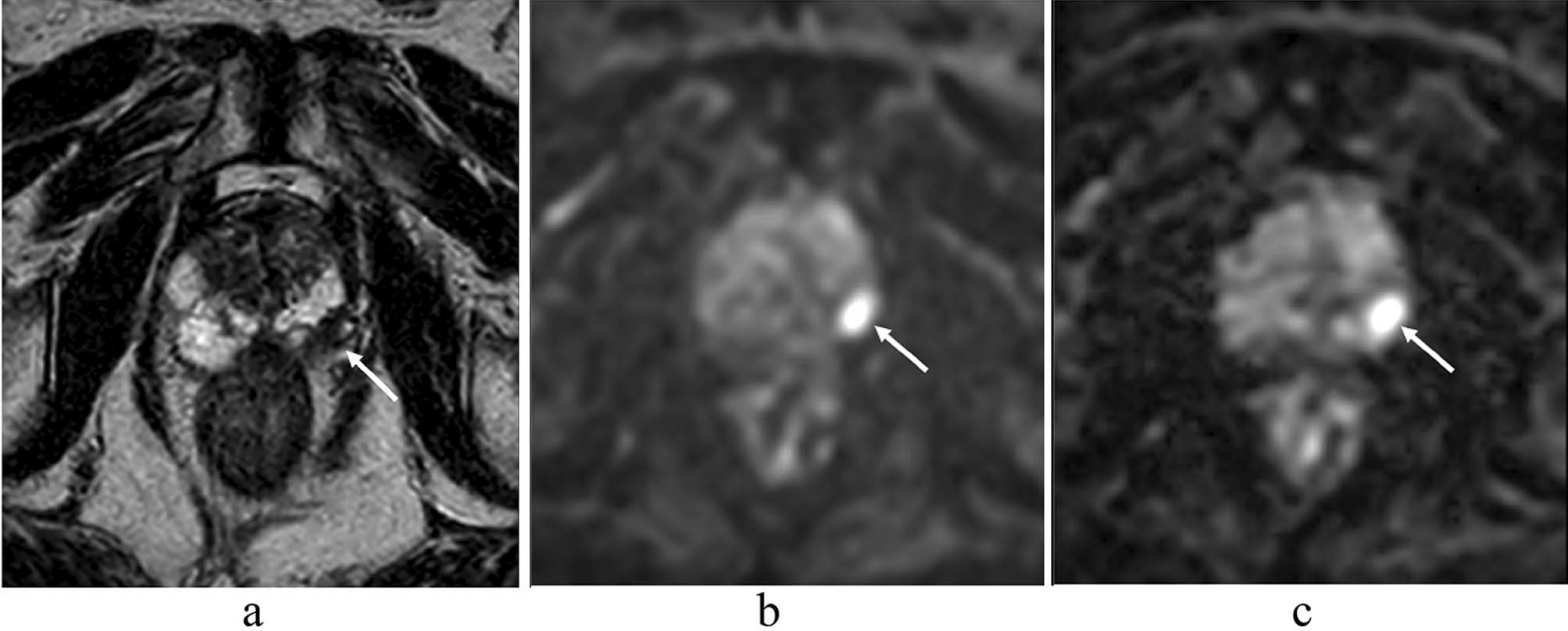
An 83-year-old man with prostate cancer (PSA level, 6.60 ng/mL; Gleason score, 3 + 4) in the left peripheral zone. A homogeneous hypointense lesion is seen on T2-weighted imaging (**a**) (arrow). A focal hyperintensity is depicted clearly on ssEPI DWI (**b**) and msEPI DWI (**c**) (arrow). The clarity of the prostate edge is better in msEPI DWI (**c**) compared with ssEPI DWI (**b**). *PSA* prostate-specific antigen, *ssEPI* single-shot echo-planar imaging, *DWI* diffusion-weighted imaging, *msEPI* multi-shot echo-planar imaging.

**Figure 3:**
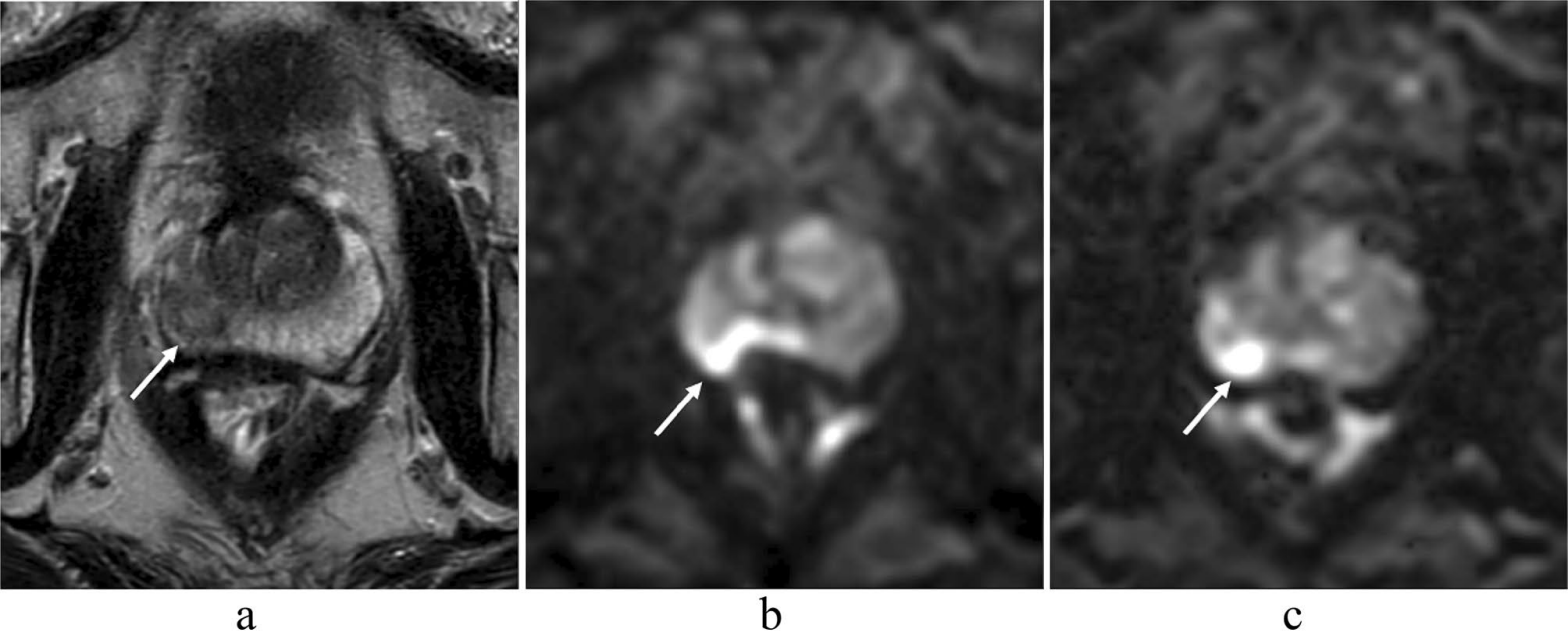
A 73-year-old man with prostate cancer (PSA level, 8.34 ng/mL; Gleason score, 3 + 3) in the right peripheral zone. A heterogeneous hypointense lesion with mass effect is seen on T2-weighted imaging (**a**) (arrow). A focal hyperintensity is depicted clearly on msEPI DWI (**c**) (arrow). Lesion clarity in ssEPI DWI (**b**) is impaired by distortion induced by rectal gas (arrow). However, SNR is higher in ssEPI DWI (**b**) than in msEPI DWI (**c**). *PSA* prostate-specific antigen, *SNR* signal-to-noise ratio, *ssEPI* single-shot echo-planar imaging, *DWI* diffusion-weighted imaging, *msEPI* multi-shot echo-planar imaging.

In the qualitative analysis, anatomical distortion and prostate edge clarity were significantly higher in msEPI DWI than in ssEPI DWI (both P < 0.001) in all three readers (Table 3) (Figs. 2 and 3), whereas quantitative SNR and CNR were significantly higher in ssEPI DWI than in msEPI DWI (SNR: 37.5 ± 13.2 and 18.0 ± 7.07, respectively, *P* < 0.001; CNR, 43.1 ± 24.0 and 18.0 ± 7.07, respectively, *P* < 0.001) (Fig. 3). There was no significant difference in lesion ADC between msEPI DWI and ssEPI DWI (0.74 ± 0.14 vs. 0.73 ± 0.14, respectively, *P* = 0.200).

**Table 3:** Comparison of qualitative indices of image quality and tumor assessment between ssEPI DWI and msEPI DWI.

	Reader 1 (9 years of experience)			Reader 2 (13 years of experience)			Reader 2 (23 years of experience)		
	ssEPI	msEPI	*p* value	ssEPI	msEPI	*p* value	ssEPI	msEPI	*p* value
Anatomical distortion	3.30 ± 0.80	3.67 ± 0.56	< 0.001	3.05 ± 0.78	3.25 ± 0.74	< 0.001	3.01 ± 0.97	3.63 ± 0.58	< 0.001
Prostate edge clarity	2.98 ± 0.48	3.73 ± 0.48	< 0.001	3.04 ± 0.67	3.55 ± 0.57	< 0.001	2.99 ± 0.67	3.48 ± 0.56	< 0.001
Lesion conspicuity score	3.70 ± 0.61	3.78 ± 0.55	0.046	3.72 ± 0.63	3.83 ± 0.38	0.107	3.61 ± 0.63	3.93 ± 0.26	0.001

### Diagnostic performance for csPC detection

**Figure 4:**
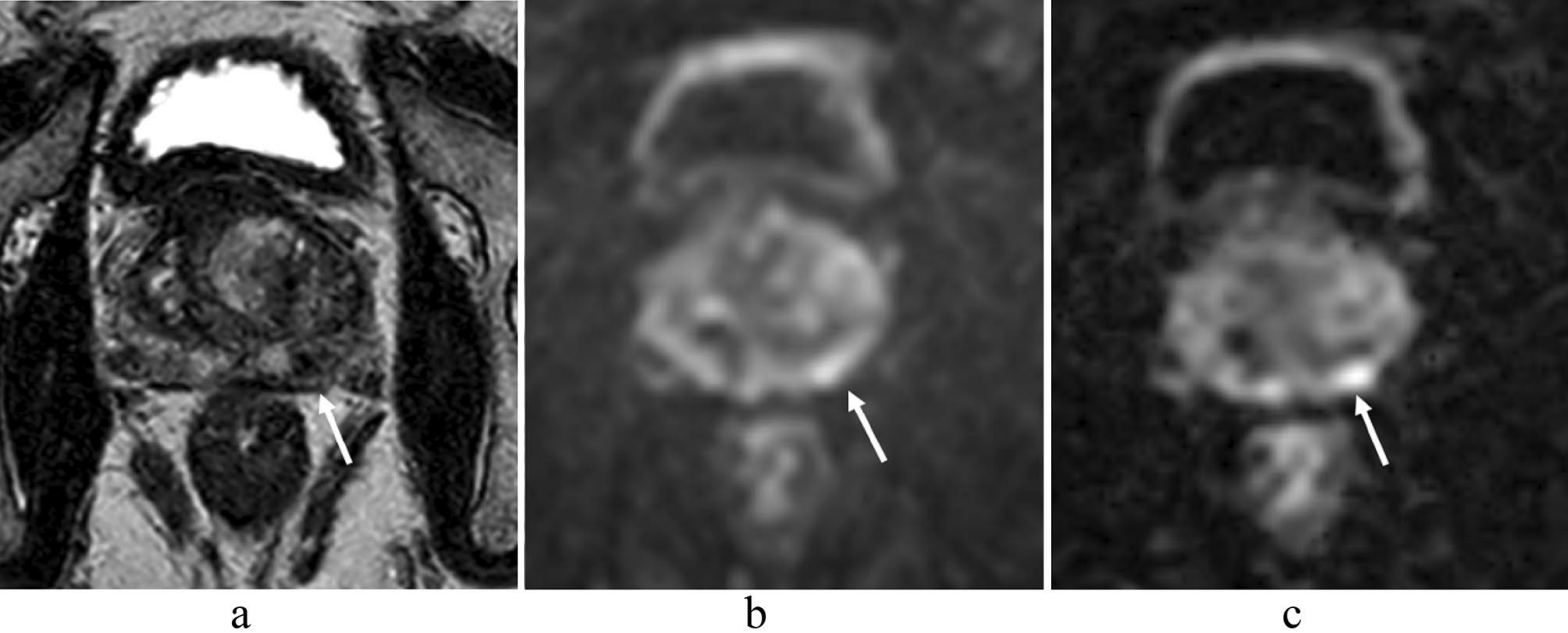
A 58-year-old man with prostate cancer (PSA level, 9.43 ng/mL; Gleason score, 4 + 3) in the left peripheral zone. A homogeneous hypointense lesion is seen on T2-weighted imaging (**a**) (arrow). A focal hyperintensity is depicted clearly on msEPI DWI (**c**) (arrow). Lesion conspicuity is lower in ssEPI DWI (**b**) than in msEPI DWI (**c**) (arrow). *PSA* prostate-specific antigen, *msEPI* multi-shot echo-planar imaging, *DWI* diffusion-weighted imaging, *ssEPI* single-shot echo-planar imaging.

In the qualitative analysis, lesion conspicuity score was significantly higher in msEPI DWI than in ssEPI DWI in reader 1 (*P* = 0.046) and reader 3 (*P* < 0.001), but not in reader 2 (*P* = 0.107) (Table 3, Figs. 3 and 4). In the quantitative analysis, lesion ADC was significantly lower than benign peripheral zone ADC in both sequences (*P* < 0.001 in ssEPI DWI and *P* = 0.008 in msEPI DWI) (Table 4).

**Table 4:** Comparison of ADC between benign and malignant tissues, and PC with GS ≤ 3 + 4 and PC with GS ≥ 4 + 3, in ssEPI DWI and msEPI DWI.

	Lesion ADC (× 10^–3^ mm^2^/s) (*n* = 55)	Normal peripheral zone ADC (× 10^–3^ mm^2^/s) (*n* = 55)	*p* value	Lesion ADC in PC with GS ≤ 3 + 4 (× 10^–3^ mm^2^/s) (*n* = 31)	Lesion ADC in PC with GS ≥ 4 + 3 (× 10^–3^ mm^2^/s) (*n* = 24)	*p* value
ssEPI	0.74 ± 0.14	1.70 ± 0.25	< 0.001	0.78 ± 0.14	0.69 ± 0.12	< 0.001
msEPI	0.73 ± 0.14	1.79 ± 0.29	0.008	0.76 ± 0.16	0.69 ± 0.11	0.029

In the analysis of 136 regions in 17 patients who underwent prostatectomy, diagnostic sensitivity, diagnostic specificity, and accuracy were higher in msEPI DWI than in ssEPI DWI in readers 1 and 3, but no significant difference was found in the overall readers for those indices (*P* ≥ 0.05 in all comparisons) (Table 5). In ROC analysis, AUC in msEPI DWI was comparable to that in ssEPI DWI in all readers (*P* ≥ 0.05 in all comparisons) (Table 5).

**Table 5:** Comparison of Diagnostic Performance for Clinically Significant Prostate Cancer Detection between ssEPI DWI and msEPI DWI.

	Reader 1 (9 years of experience)			Reader 2 (13 years of experience)			Reader 2 (23 years of experience)		
	ssEPI	msEPI	*p* value	ssEPI	msEPI	*p* value	ssEPI	msEPI	*p* value
Sensitivity	72.1 (49/68)	75.0 (51/68)	0.500	60.3 (41/68)	63.2 (43/68)	0.500	69.1 (47/68)	73.5 (50/68)	0.625
Specificity	89.7 (61/68)	92.6 (63/68)	0.625	92.6 (63/68)	88.2 (60/68)	0.250	76.5 (52/68)	86.8 (59/68)	0.065
Positive predictive value	87.5 (49/56)	91.1 (51/56)	NA	89.1 (41/46)	84.3 (43/51)	NA	74.6 (47/63)	84.7 (50/59)	NA
Negative predictive value	76.3 (61/80)	78.8 (63/80)	NA	70.0 (63/90)	70.6 (60/85)	NA	71.2 (52/73)	76.6 (59/77)	NA
Accuracy	80.9 (110/136)	83.8 (114/136)	0.219	76.5 (104/136)	75.7 (103/136)	1.000	72.8 (99/136)	80.1 (109/136)	0.057
AUC	0.844	0.850	0.605	0.774	0.781	0.632	0.787	0.821	0.097

Data (excluding AUC) are presented as percentages, with values used to calculate percentages in parentheses.

**Figure 5:**
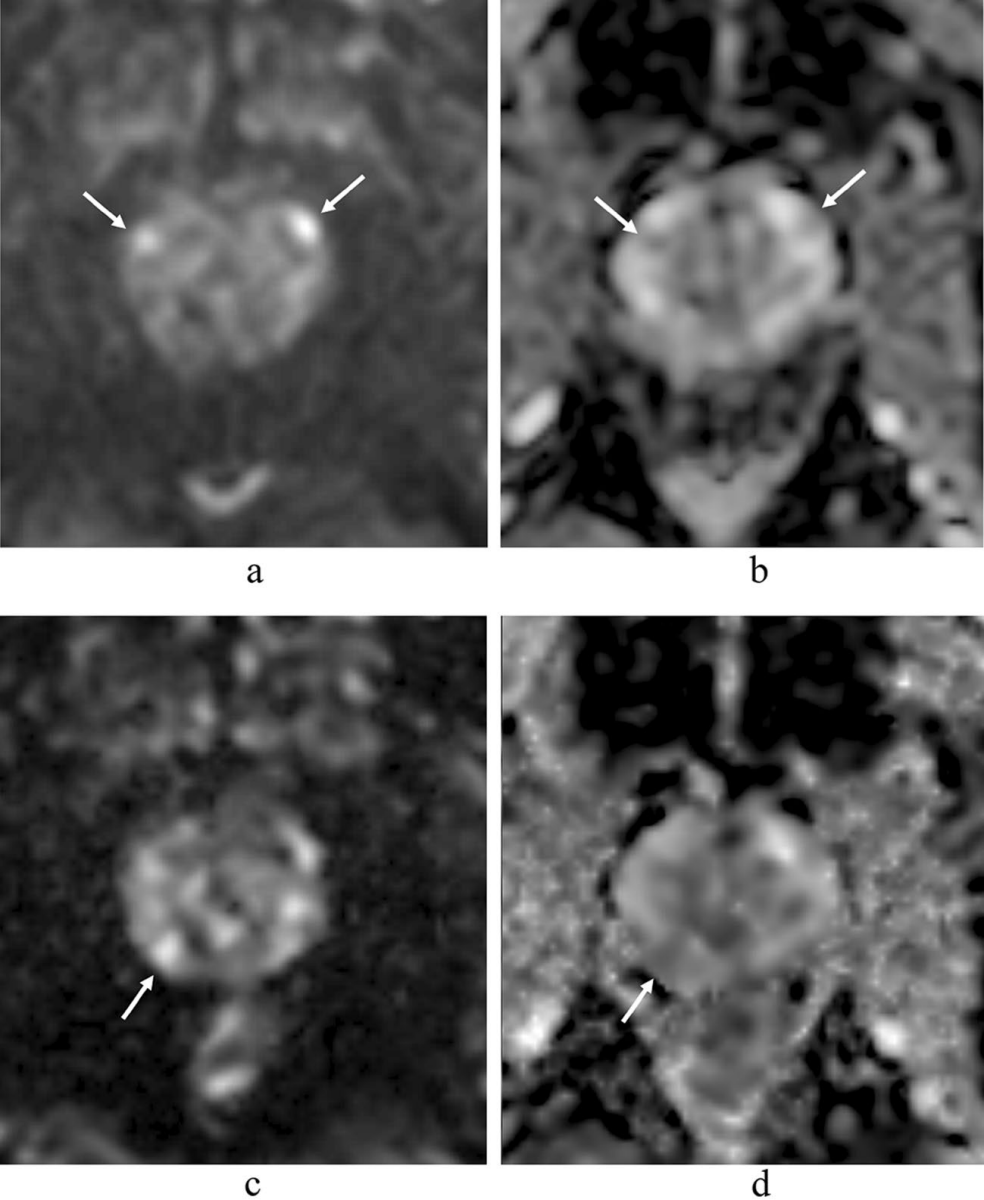
A 77-year-old man with prostate cancer (prostate-specific antigen level, 5.10 ng/mL; Gleason score, 4 + 3) in the apex of the left peripheral zone and the left transition zone (not shown). SsEPI DWI (**a**) and ADC map in ssEPI DWI (**b**) show lesions corresponding to a DWI/ADC map score of 3 on the anterior middle regions of the bilateral peripheral zones (arrows). However, the same regions were diagnosed with a DWI/ADC map score of 1 in msEPI DWI (**c**) and ADC map in msEPI DWI (**d**). MsEPI DWI (**c**) and ADC map in msEPI DWI (**d**) show a lesion corresponding to a DWI/ADC map score of 2 in the posterior middle region of the right peripheral zone (arrow). Prostatectomy specimens confirmed a diagnosis of benign prostatic condition for the three middle regions in the right and left peripheral zones. Thus, for the bilateral middle peripheral zone lesions, ssEPI DWI gave a false-positive result and msEPI DWI showed a true-negative result. *PSA* prostate-specific antigen, *ssEPI* single-shot echo-planar imaging, *DWI* diffusion-weighted imaging, *ADC* apparent diffusion coefficient, *ssEPI* single-shot echo-planar imaging.

In all readers, there were 26 regions with discrepant results for csPC detection between the sequences: 8 were true positive by msEPI DWI and false negative by ssEPI DWI, 11 were true negative by msEPI DWI and false positive by ssEPI DWI, 6 were false positive by msEPI DWI and true negative by ssEPI DWI, and one was false negative by msEPI DWI and true positive by ssEPI DWI (Fig. 5).

### Diagnostic performance of lesion ADC for tumor aggressiveness in PC

Lesion ADC in PC with GS ≥ 4 + 3 was significantly lower than that in PC with GS ≤ 3 + 4 in both sequences (0.69 ± 0.12 vs. 0.78 ± 0.14, *P* < 0.001 in ssEPI DWI and 0.69 ± 0.11 vs. 0.76 ± 0.16, *P* = 0.029 in msEPI DWI) (Table 4). Regarding discrimination ability between PC with GS ≤ 3 + 4 and PC with GS ≥ 4 + 3 using lesion ADC, the AUC was comparable between ssEPI DWI and msEPI DWI (0.711 and 0.673, respectively, *P* = 0.314).

## Discussion

Using MR-guided biopsy with and without prostatectomy as the reference standard, we compared image quality and diagnostic performance for csPC detection between ssEPI DWI and msEPI DWI in 134 male patients.

MsEPI DWI such as IRIS is sensitive to phase error caused by motion during application of the motion-probing gradient because it is a multi-shot acquisition. The motion generates phase errors that vary from shot to shot, resulting in ghost artifacts in the reconstructed image. However, there were no cases in this study that could not be diagnosed due to motion artifacts, thanks to 2D navigator echo. In comparison of quantitative image quality, SNR and CNR were higher in ssEPI DWI than in msEPI DWI under the same scan time. This is related to the fact that the number of signal averages was doubled in ssEPI DWI compared with msEPI DWI (number of shots: 2) to keep the scan time the same between the two sequences, and increased SNR leads to a higher CNR. Conversely, as expected, measures of image distortion and image sharpness such as anatomical distortion and prostate edge clarity showed better results in msEPI DWI than in ssEPI DWI in all readers. Regarding the application of msEPI DWI to prostate imaging, a recent study using RESOLVE at 3.0 T has reported that subjective image quality (taking into account artifacts, delineation of anatomic structures and borders, overall sharpness, contrast, and overall subjective impression) and CNR of PC and benign prostatic tissue were all higher for msEPI DWI than for ssEPI DWI, but that SNR was lower for msEPI DWI than for ssEPI DWI^37^. As the acquisition times in that study were 7 min 33 s for msEPI DWI and 3 min 54 for ssEPI DWI, a possible reason for the difference in CNR compared with the present result may be related to the difference in TE. In our study, all imaging parameters other than the EPI factor (and the associated number of shots) and number of signal averages were set as the same, whereas in the previous study, TE was quite different between ssEPI and RESOLVE (88 ms and 55 ms). It is well known that the T2 of PC is shorter than that of normal prostate tissue^38–40^. Therefore, use of a shorter TE in prostate DWI could improve diffusion contrast by reducing T2 shine-through effect due to the longer T2 of normal prostate tissue. It should be noted that in our study, msEPI DWI contributed to improvement of image quality (excluding SNR and CNR) in a clinically applicable imaging time of 4 min 24 s using the minimum number of shots (2 shots). Nevertheless, the inferior SNR in msEPI DWI compared with ssEPI DWI did not affect improvement of image quality.

In comparison of tumor detection ability in csPC, qualitative lesion conspicuity score was higher in msEPI DWI than in ssEPI DWI, and the difference was statistically significant in two of the three readers. The reduction of distortion, blurring, and ghost artifacts in msEPI DWI (which acquires k-space data with shorter shot length in multiple excitations and provides robust image quality by minimizing motion effects during diffusion encoding between shots using 2D navigator echo) may be the reasons for improved lesion conspicuity of csPC with this sequence^30,31^. Regarding the diagnostic performance of csPC detection using the DWI score of PI-RADS v2.1 in PC patients who underwent prostatectomy, diagnostic sensitivity, diagnostic specificity, accuracy, and AUC were comparable between msEPI DWI and ssEPI DWI in all three readers. However, in the discrepant results in the total of 26 regions in three readers between the two sequences, 19 regions were correct by msEPI DWI and incorrect by ssEPI DWI, and 7 regions were incorrect by msEPI DWI and correct by ssEPI DWI. These findings indicate that msEPI DWI has equivalent or better diagnostic performance compared with ssEPI DWI. All patients received intramuscular injections of scopolamine butylbromide or glucagon to minimize intestinal peristalsis, which is a routine prostate MRI protocol at our hospital. Therefore, at hospitals where such drugs are not administered, msEPI DWI has potential to improve image quality and diagnostic performance. In addition, the significance of image quality and csPC detection ability in msEPI DWI compared with ssEPI DWI may be particularly useful for less experienced radiologists. The discrimination ability between PC and benign prostatic tissue using quantitative lesion ADC in msEPI DWI was similar to ssEPI DWI, which has already been reported to have high diagnostic performance^41^.

In the assessment of tumor aggressiveness in PC, for discrimination ability between PC with GS ≤ 3 + 4 and PC with GS ≥ 4 + 3 using quantitative lesion mean ADC, lesion ADC was lower in PC with GS ≥ 4 + 3 than in PC with GS ≤ 3 + 4 for both sequences, and AUC was comparable between ssEPI DWI and msEPI DWI. The AUC values (0.711 and 0.673, respectively) in our study correspond to those reported in previous studies^41^.

Our study had several limitations. First, this was a single-institutional retrospective study with a relatively small number of patients. It is necessary to validate the present results in future prospective multi-institutional clinical studies with multiple readers and a larger number of patients. Second, because distortion and blurring were reduced in msEPI DWI compared with ssEPI DWI, we would expect msEPI DWI to contribute to improving diagnostic performance in local staging for cases such as extracapsular extension. However, as only 17 of the present patients underwent prostatectomy, there was insufficient statistical power to withstand this analysis. Third, the intra-scan reproducibility of ADC for mshEPI and sshEPI was not performed. In order to accurately observe the ADC, the reproducibility is of significance. Further validation will be needed for quantification of ADC. Finally, the acquisition time of ssEPI DWI was about twice that used in daily clinical practice, and is thus not comparable with conventional ssEPI DWI. However, as a first trial in comparison between ssEPI DWI and msEPI DWI, we focused on using the same acquisition time for both sequences to remove the limitation of potential extension of acquisition time in msEPI DWI compared with ssEPI DWI. Furthermore, the longer acquisition time in msEPI DWI compared with ssEPI DWI can be improved by reduced number of signal averages with imaging noise reduction techniques such as deep-learning-based reconstruction.

In conclusion, the present findings suggest that IRIS, a msEPI DWI sequence, shows improved image distortion and blurring compared with ssEPI DWI and may contribute to increased diagnostic performance of csPC detection compared with ssEPI DWI under the same scan time. msEPI DWI acquired with a scan time of 4 min 24 s may be acceptable in daily clinical practice for detecting csPC in patients with elevated PSA levels and could be an alternative to ssEPI DWI.

## Data Availability

The datasets used and/or analyzed during the current study available from the corresponding author on reasonable request.
